# Genotyping and pathogenicity of fowl adenovirus isolated from broiler chickens in Egypt

**DOI:** 10.1186/s12917-022-03422-1

**Published:** 2022-08-30

**Authors:** Marwa M. Safwat, Al Shimaa R. Sayed, Marwa F. Ali Elsayed, Awad Abd El Hafez Ibrahim

**Affiliations:** 1 Department of Avian and Rabbit Medicine, Faculty of Veterinary Medicine, Assiut University, Assiut, Egypt; 2 Department of Poultry Diseases, Agriculture Research Center, Animal Health Research Institute, Assiut Lab, Egypt; 3 Department of Pathology and Clinical Pathology, Faculty of Veterinary Medicine, Assiut University, Assiut, Egypt

**Keywords:** Fowl adenovirus serotype 2, Inclusion body hepatitis, Pathogenesis, Histopathological examination

## Abstract

**Background:**

Over the past 10 years, inclusion body hepatitis outbreaks, essentially from commercial broiler flocks, have been detected in different geographic regions highlighting the wide distribution of FAdVs around the world resulting in serious economic losses due to increased mortalities as well as poor performance within poultry farms in Assiut province, Egypt. Thus, this study was achieved to detect fowl adenovirus in broiler chicken flocks in Assiut province, Egypt and to recognize the pathogenicity of the isolated virus.

**Results:**

The phylogeny of the L1 loop of the hexon gene exposed that the isolated virus clustered and belonged to the reference strains serotype D FAdV. The isolated virus is closely related to inclusion body hepatitis (IBH) strains causing extensive economic losses. The pathogenicity study of the virus showed typical macroscopic lesions with 6% mortality; furthermore, histopathological inspection exhibited severe hepatitis and degenerative changes after 5d from infection in the immune system.

**Conclusion:**

Results in this research support the primary pathogenicity and mortality caused by FADV serotype 2 (IBH) alone without immunosuppressive agents thus robust control measures should be implanted against FAdV to evade the serious economic losses in poultry farms.

**Supplementary Information:**

The online version contains supplementary material available at 10.1186/s12917-022-03422-1.

## Background

Fowl adenoviruses (FAdVs) are double stranded DNA-viruses and have no envelope classified as member of Aviadenovirus genus within Adenoviridae family. On molecular basis restriction fragment length polymorphism (RFLP) were divided into 5 species from A to E [1], and classified into 12 serotypes from 1to 8a—8b and 11 by neutralization assay [2]. Inclusion body hepatitis (IBH), hepatitis-hydro pericardium syndrome (HHS) and gizzard erosions (GE) are the most common diseases in broiler chickens associated with FAdV infection [3].

IBH is a severe disease majority of its occurrence in young broilers with age ranging from 3 to 7 weeks and caused by multiple serotypes of FAdV [4]. Fowl adenovirus Serotypes 2, 3, 9, and 11 of species D and 6, 7, 8a, and 8b serotypes of species E are main cause of IBH [5–7]. The most of observed outbreaks caused by serotypes 2, 4, 8a, 8b and 11 as reported in New Zealand [8], Canada [9], Japan [10], Australia [6], Korea [11], Hungary [12], South Africa [13], China [14], and recently Saudi Arabia [15] and Egypt [16, 17].

IBH has Characterized curve of mortality reach to peak within 3-4 days of infection and return to normal on 5–6 days of infection, usually mortality percent ranges from 5 into 10% [3] and sometimes reaches to 30% [18]. While rate of morbidity is low and diseased chickens have adopt a crouching position with ruffled feathers [3, 19]. At postmortem examination, the liver of diseased birds is pale, friable, swollen and petechial hemorrhages may be present in skeletal muscle [19].

Diagnosis of IBH has based on observation macroscopic lesions along with histopathological lesions in examined birds. Macroscopically, affected birds usually show pale yellow, friable and swollen livers, also petechial and/or ecchymotic haemorrhages may be present in liver and muscles [19]. Two kinds of Intra nuclear inclusion bodies (INIB) are often detected in degenerated hepatocytes which can be large round or irregularly shaped with a clear pale halo eosinophilic or basophilic inclusions occupying the nucleus [20, 21].

FAdVs has been molecularly characterized by polymerase chain reaction (PCR) using specific hexon gene primers which is the most common gene used for detection FAdVs [22, 23]. Also primer specific for DNA polymerase [24], in addition to DNA sequencing and/or restriction enzyme analysis has been used for FAdV typing [22, 25], Although hexon is the major protein of the adenovirus that possess the neutralizing epitope, and known to be serotype specific [26, 27], which mean that serotyping of FAdV is mainly related to sequencing of the hexon gene [28]. So the aim of this study knowledge Genotyping of Adenovirus associated with inclusion body hepatitis in infected broiler chickens in Assiut province, Egypt and also its Pathological effect.

## Methods

### Samples collection

One hundred Liver tissues were collected from ten poultry broiler farms as pooled samples (ten per each flock) suspected to be inclusion body hepatitis, based on clinical signs and postmortem examination. Affected birds showed depression, decreased body weight, and watery diarrhea with age ranged from 28-37 days. The most prominent gross lesions were pale, swollen livers with sub capsular ecchymotic hemorrhages. liver samples were divided into two parts one part was kept in 10% neutral buffered formalin (NBF) for histopathological examination and an another part was processed for virus isolation as follow, Fragments of liver samples were aseptically homogenized in phosphate buffered saline (PBS) 10% containing 200U/ml penicillin and 0.2 mg/ml streptomycin. Liver tissues were centrifuged for 10 min at 2000 rpm [29]. A reverse transcriptase-PCR was done to approve that the liver homogenates of field samples free from contamination with LPAI-H9N2, NDV and IBD viruses. Supernatants were transferred to fresh sterile tubes and conserved at − 40˚C until further use.

### Histopathological examination

Neutral Buffer Formalin-fixed liver tissues (4 μm sections) were embedded in paraffin according to standard methods and stained with hematoxylin and eosin for microscopic changes examination [30].

### Isolation of Fowl adenovirus (FAdV) in embryonated chicken eggs

Isolation was attempted on 9^th^ -day old SPF embryonated chicken eggs (ECEs) with 2 successive passages by inoculating 0,2 ml of liver tissue supernatant through allantoic route into five ECEs per each sample. Five ECEs, inoculated with phosphate buffer saline (PBS), were considered as a negative control. Incubation of eggs was at 37˚C for 10 days and candled daily. Embryos which died after 24 h post-inoculation were culled, other than those that survived until the end of experiment were harvested for molecular detection of IBH virus by PCR.

### DNA extraction

Aliquots (200 μl) of supernatant of homogenized liver tissues and allantoic fluids were used for viral DNA extraction of using thermo scientific kit (Nucleospin Tissue, Germany) according to the manufacturer’s instructions. Elution of DNA was in 70 μl nuclease-free water, and 4 μl of viral DNA was used for PCR template.

### Primers set

Primers targeted a conserved region in the L1 region of the hexon gene, designated by [31] that supposedly yield a 590 bp amplicon;

Hex L1-s 301–323 5-ATGGGAGSACCTAYTTCGACAT-3 and Hex L1-as 890–868 5-AAATTGTCCCKRAANCCGATGTA-3.

### Polymerase chain reaction (PCR)

PCR was done as the following: 1 cycle at 95 °C for 5 min; followed by 35 cycles at 94 °C for 45 s, 51 °C for 45 s, and 72 °C for 45 s; and then, a final extension at 72 °C for 10 min. PCR Amplicons were visualized by gel electrophoresis according to [32].

### Partial hexon gene sequencing

DNA bands with 590 bp size (all positive samples) were excised from the gel and the PCR amplicon was extracted using Gene JET Gel Extraction kit (Thermo scientific, Lithuania) according to the manufacturer instructions. Direct Sanger sequencing method was performed using the forward and reverse primers in one reaction with the purified DNA fragment.

### Phylogenetic analysis

Edition and comparison of FAdV sequences with other published sequences available in GenBank, using BLAST tool of the National Center for Biotechnology Information NCBI, were performed. Sequences were aligned using the Clustal W program. Phylogenetic tree was set using the Neighbor-Joining MEGA program version 6. The stability of relationships was performed by bootstrapping analyses of N-J data based on 500 re samplings.

#### Pathogenicity of the isolated virus

##### Virus titration

The titer was determined by Veterinary Serum and Vaccine Research Institute (VSVRI), Abbassia, Cairo. Confluent monolayers of chicken embryo liver (CEL) cells prepared from 16d old specific pathogen free embryos were used for inoculation of liver supernatants according to [33]. The supernatant was collected and stored at -40C.

##### Experimental study

One hundred White Leghorn Layer chicks were received at one day old and kept in experimental units of Avian and Rabbit Medicine Department, Faculty of Veterinary Medicine, Assiut University approved by The National Ethical Committee of The Faculty of Veterinary Medicine, Assiut University, Assiut, Egypt, according to The OIE standards for use of animals in research in accordance with ARRIVE guidelines to study the pathogenicity of the FAdV. At 5^th^ day of age, chicks were roughly divided in 2 groups: 30 birds in the control group and 70 in the test group. Birds were infected with 10 ^6^ 50% tissue culture infective dose (TCID50)/ml [16] through oro-pharyngeal route and observed daily for 40 days after FAdV isolated strain (OK482670) challenge. Signs and lesions were observed At 3, 5, 7, 9 and 35 days post infection (dpi), 3 birds from the test group were euthanized; liver, bursa and lung were collected. Portions of liver samples were pooled and preserved at—40^*◦*^C for molecular detection using PCR.

##### Histopathological examination

Fresh specimens from liver, bursae and lung of chicken from 3 birds at 3, 5, 7, 9, and 35 day post infection were collected and fixed in 10% neutral-buffered formalin. The tissues were dehydrated in a graded alcohol series, cleared with methyl benzoate, embedded in paraffin wax, sectioned at 4-μm thickness and stained with haematoxylin and eosin for histopathological examination by light microscopy (Olympus CX31, Japan) and photographed using digital camera (Olympus, Camedia C-5060, Japan) [30].

##### Transmission electron microscopy (TEM)

Liver of chicken from 3 birds at 5 day post infection was fixed in 5% glutaraldehyde and approximately 1 × 1x1mm blocks were prepared. Blocks were washed in cacodaylate buffer (0.1 M, pH 7.2) for three times (20 min each) and then post- fixed in 1% osmium tetraoxide for 2 h, dehydrated in ascending grades of ethyl alcohol up to 100% (30 min for every concentration), and embedded in epon. Semi thin sections were obtained at 1 µ by using LKB ultratome and stained with toluidine blue and examined by light microscope. Ultrathin Sects. (70 nm) were cut using a diamond knife (Reichert OMU3 ultramicrotome).The thin sections were mounted on copper grids (200 mesh) and double stained with uranyl acetate and lead citrate. The ultrastrucural examination was carried out by using a transmission electron microscope (Jeol, CXII) at 80 kv (Electron Microscope Unit, Assiut University).

## Results

### Macroscopic and microscopic lesions

**Fig. 1:**
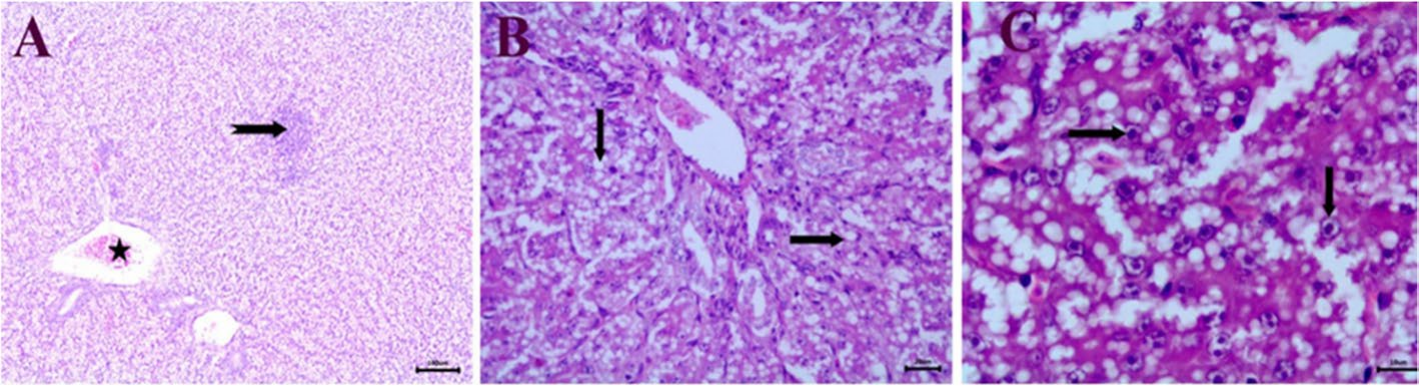
Histopathological examination of infected birds with fowl adenovirus from survey study: **A**, **B** Liver showing focal infiltration of mononuclear inflammatory cells (notched arrow), congestion of central vein (star), lytic necrosis of hepatic cells (arrow) (bar = 20), **C** presence of intra nuclear basophilic inclusion bodies in hepatocytes (arrow) (bar = 10), (H&E)

Grossly affected birds had swollen and pale friable liver with pin point ecchymotic hemorrhage and sub capsular hemorrhage, microscopically obtained field liver samples of suspected cases showed characteristic histopathological lesions of fowl adenovirus such as focal infiltration of mononuclear inflammatory cells and congestion of central vein (Fig. 1A) and lytic necrosis of hepatic cells (Fig. 1B), appearance of basophilic Intranuclear inclusion bodies in hepatocytes (Fig. 1C).

### Isolation of FAdV in embryonated chicken eggs

**Fig. 2:**
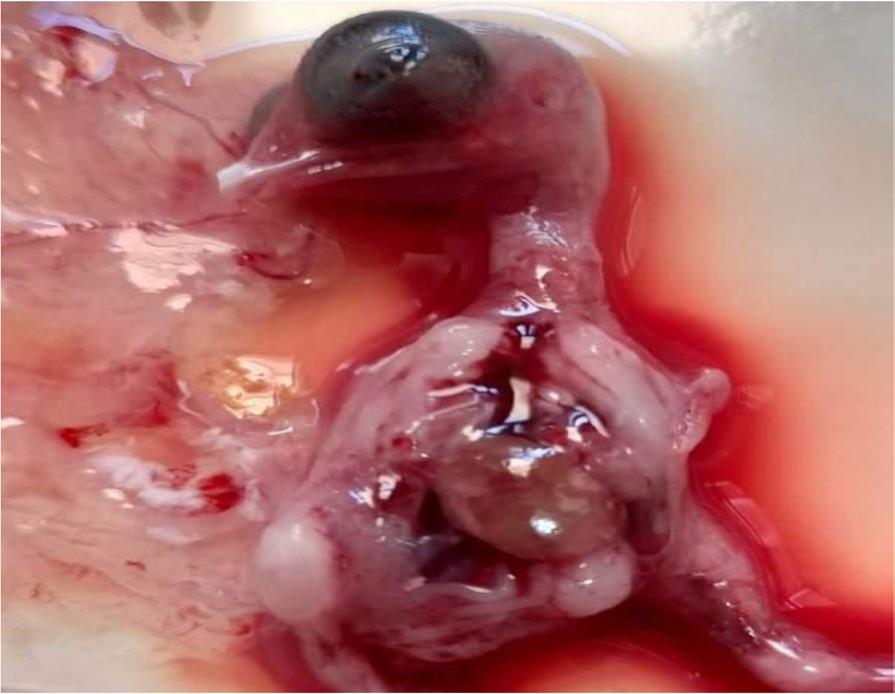
Embryo inoculated with FAdV showing enlarged friable liver with reddish and necrotic foci and thickening in the CAM

Not all embryos died after 5 days post inoculation with liver homogenates of affected chicken farms. Some of them were alive till the 10^th^ day post inoculation and embryos were hemorrhagic, and enlarged friable livers with yellow to reddish necrotic foci and/or diffuse greenish discoloration and thickening in the chorioalantoic membrane were observed (Fig. 2).

### Molecular detection of FAdV by PCR

**Fig. 3:**
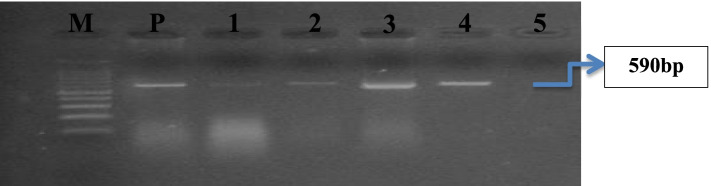
Showing M; DNA marker, P positive control, 1, 2, 3 and 4 positive samples give band with molecular weight 590 bp and 5 negative sample

Confirmation of presence FAdV by PCR amplicon size of loop1 region of hexon gene (590 bp) by gel electrophoresis in 4 out of 10 commercial broiler farms (40%) (Fig. 3).

### Sequence and phylogenetic analysis

**Fig. 4:**
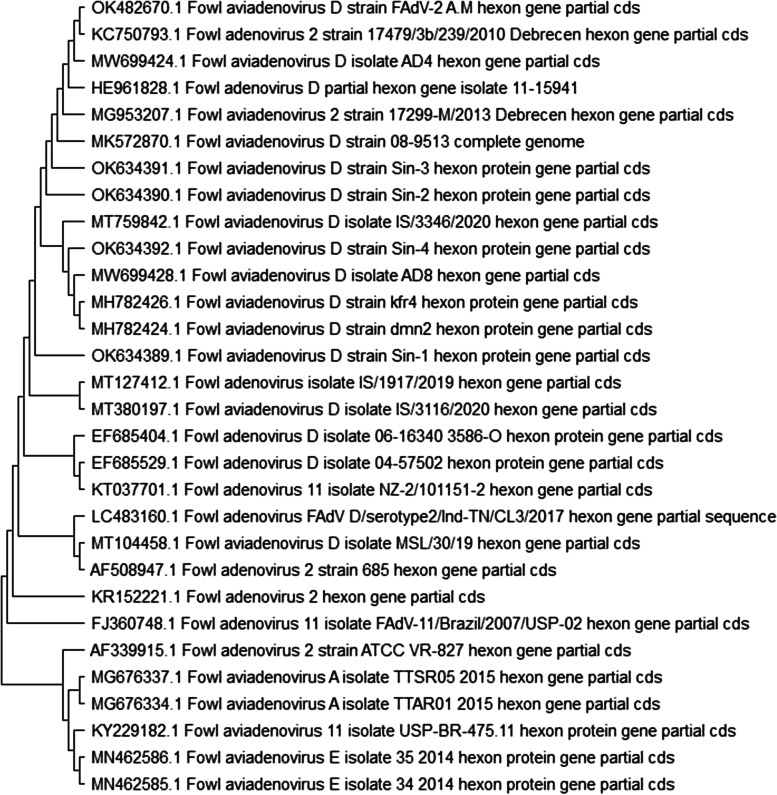
Phylogenetic analysis of FAdV strain (OK482670) with other FAdV isolated from different countries with using Mega X program

Sequencing of our isolates of FAdV partial hexon gene was analyzed with those sequences from different countries available on GenBank and it is genetically related to FAdV species D serotype 2(OK482670). The phylogeny analyzed that FAdV strain of this study is closely related to FAdVs from Hungary, Egypt, Austria, Japan and Israel (Accession numbers KC750793, MW699424, HE961828, MK572870 and MT759842) (Fig. 4), with identity percentages ranged from 98.9% to 99.2% at nucleotide level.

### Pathogenicity of isolated virus

**Fig. 5:**
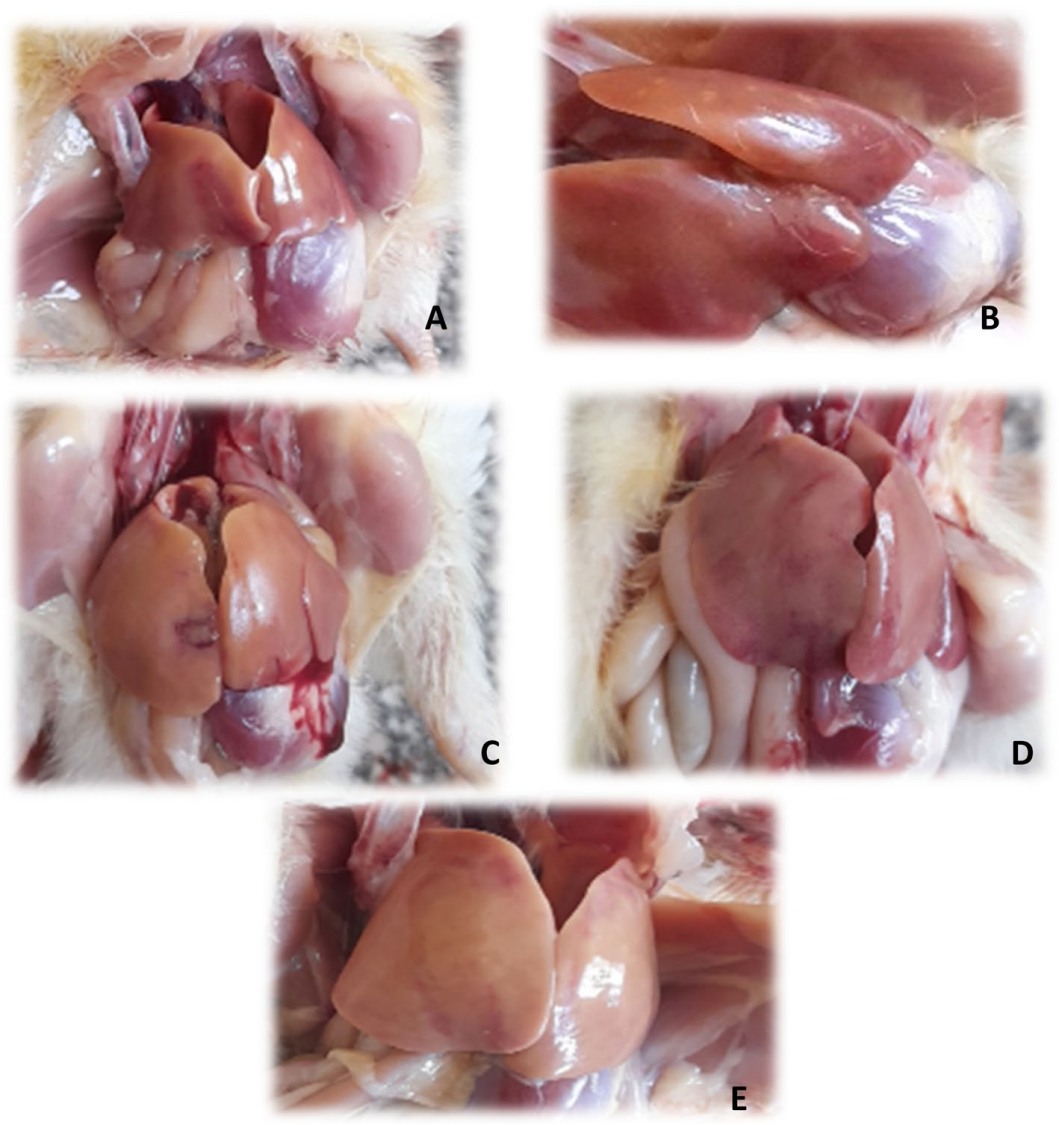
**A** Hemorrhages on liver surface, **B** focal necrosis in liver (white pin head or red foci), **C** focal necrosis in liver with subscapular haemorrhages, **D** mosaic appearance of liver, **E** hemorrhage on liver surface with focal necrosis

***Gross Pathology:*** at 3^rd^ dpi the most prominent lesion was observed in the liver, which was green discoloration enlarged liver with hemorrhages in most necropsied birds, while in others it was pale and fatty. At 5^th^ dpi, severe hepatitis and several pinhead white or red foci were observed on necropsy persisted to the end of experiment with 6% mortality (Fig. 5) with mosaic appearance and clear fine edges of the liver. All control birds remained clinically normal during the period of observation prior to death.

### Histopathology

**Fig. 6:**
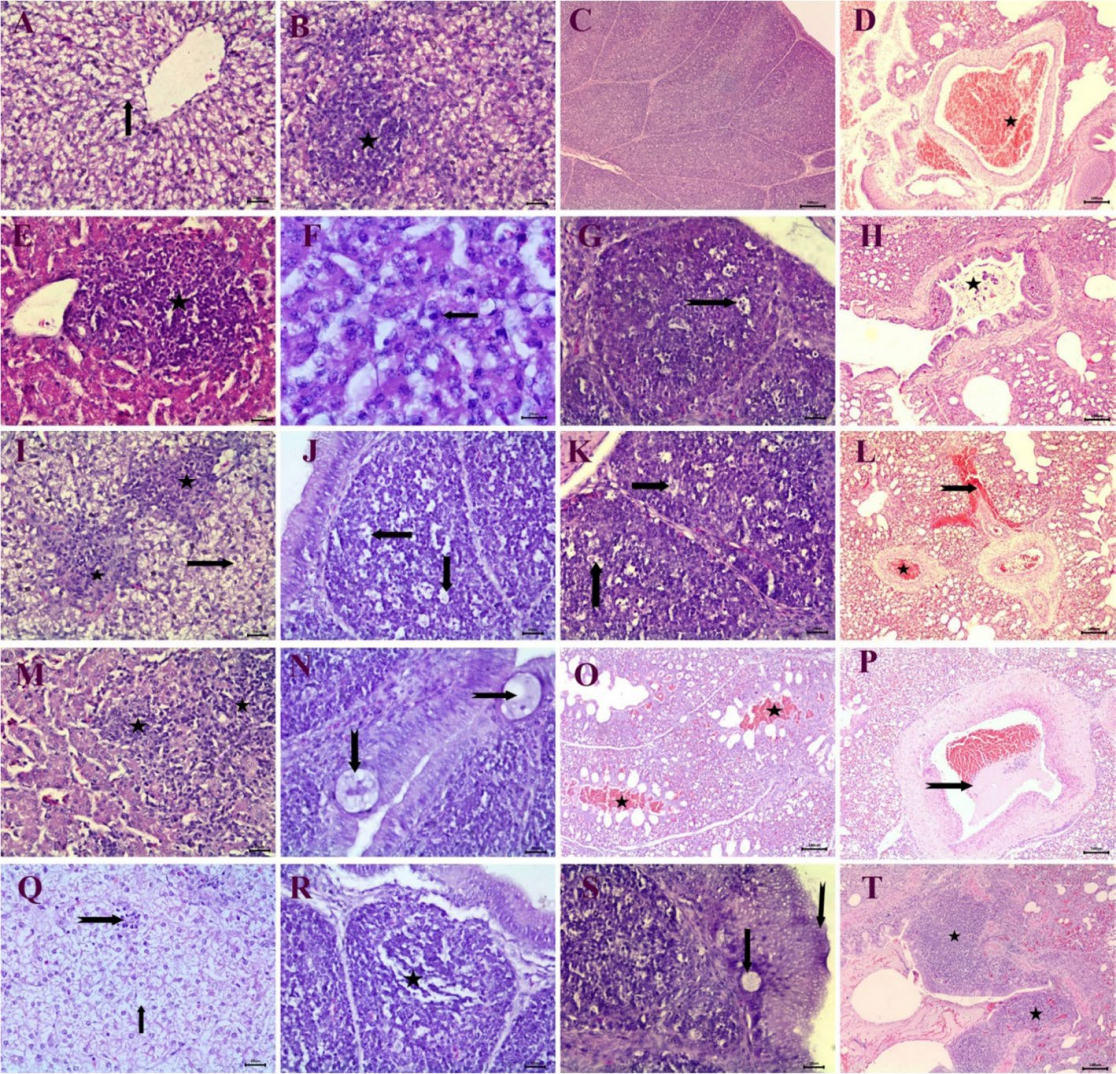
Histopathological examination of experimental birds infected with fowl adenovirus: **A** liver at 3 days post infection showing vacuolar degeneration of hepatocytes (arrow) (bar = 20), **B** liver at 3dpi showing focal infiltration of inflammatory cells (bar = 20), **C** bursa at 3dpi has no changes (bar = 100), **D** lung at 3dpi showing congestion of blood vessels (star) (bar = 100), **E**, **F** liver at 5dpi showing infiltration of inflammatory cells (star) (bar = 20), appearance of intranuclear basophilic inclusion bodies in hepatocytes (arrow) (bar = 10), **G** bursa at 5dpi showing severe lymph cytolysis (notched arrow) (bar = 20), **H** lung at 5dpi showing catarrhal exudates in the bronchi (star) (bar = 100), **I** liver at 7dpi showing inflammatory cells infiltration (star), vacuolar degeneration of hepatocytes (arrow) (100). **J** and **K** Bursa at 7dpi showing necrosis of medullary lymphocytes (arrow) (bar = 20). **L** Lung at 7dpi showing perivascular hemorrhage (notched arrow), vascular congestion (star) (bar = 100). **M** Liver at 9dpi showing severe infiltration of inflammatory cells (star) (bar = 20). **N** Bursa at 9dpi showing epithelial cysts (notched arrow) (bar = 20). **O**, **P** Lung at 9dpi showing hemorrhagic exudate in the parabronchi lumen (star), thrombosis in some blood vessels (notched arrow) (bar = 100). **Q** Liver at 35dpi showing severe lytic necrosis of all hepatic tissue (arrow) focal infiltration of inflammatory cells (notched arrow) (bar = 20). **R**,**S** Bursa at 35 days post infection showing severe medullary exhaustion of lymphocytes in lymphoid follicles (star), Hyperplasia of the bursal epithelium (notched arrow), epithelial cyst formation (arrow) (bar = 20). Lung at 35dpi showing peribronchial lymphoid hyperplasia (star) (bar = 20), (H&E)

Histopathological examination of liver at 3 days post infection showed vacuolar degeneration of most hepatic tissue (Fig. 6A) and focal infiltration of mononuclear inflammatory cells (Fig. 6B). Bursa of experimental birds had no changes after 3 days of infection (Fig. 6C). Congestion of numerous blood vessels was also observed in lung of birds at 3 days post infection (Fig. 6D).

Infiltration of mononuclear inflammatory cells and heterophilis was also detected in liver after 5 days of infection (Fig. 6E). Intranuclear basophilic inclusion bodies also began to be noticed in hepatic tissue (Fig. 6F). Birds were examined 5dpi showed diffuse vacuolization of the lymphoid follicles beside the damaging process of medullary lymphocytes expressed by severe lymphocytolysis and sign of sporadic apoptotic cells (Fig. 6G). Lung of birds in the same group revealed acute bronchopneumonia with catarrhal exudates in the bronchial lumen (Fig. 6H).

In liver, examined 7dpi, the lesions consisted of multiple focal areas of infiltration of inflammatory cells associated with vacuolar degeneration of hepatic tissue (Fig. 6I). The damaging process of medullary lymphocytes in bursa was still also observed with disappearance of reticuloepithelial layer (Fig. 6J and K). Angiopathic lesions were expressed in lung by perivascular hemorrhage and congestion of multiple blood vessels (Fig. 6L).

The results of histopathological examination of birds sacrificed 9 days post infection characterized by severe infiltration of inflammatory cells in hepatic tissue (Fig. 6M). Bursal tissues were examined 9dpi demonstrated microscopic changes similar to the above mentioned lesions. Furthermore, multiple epithelial cysts were seen (Fig. 6N). Severe lesions were detected in lung of birds 9 days post infection characterized by presence of hemorrhagic exudate in the parabronchi lumen (Fig. 6O). Thrombus formation in some blood vessels was also observed (Fig. 6P).

Histopathological examination of liver at 35dpi revealed severe lytic necrosis of all hepatic tissue with focal infiltration of inflammatory cells (Fig. 6Q). In bursa of birds in this group showed severe medullary exhaustion of lymphocytes in most lymphoid follicles (Fig. 6R). Hyperplasia of the bursal epithelium with epithelial cyst formation was evident findings (Fig. 6S). Peribronchial lymphoid hyperplasia appeared in lung of some birds after 35dpi (Fig. 6T).

### Transmission electron microscopic examination

#### Liver

**Fig. 7:**
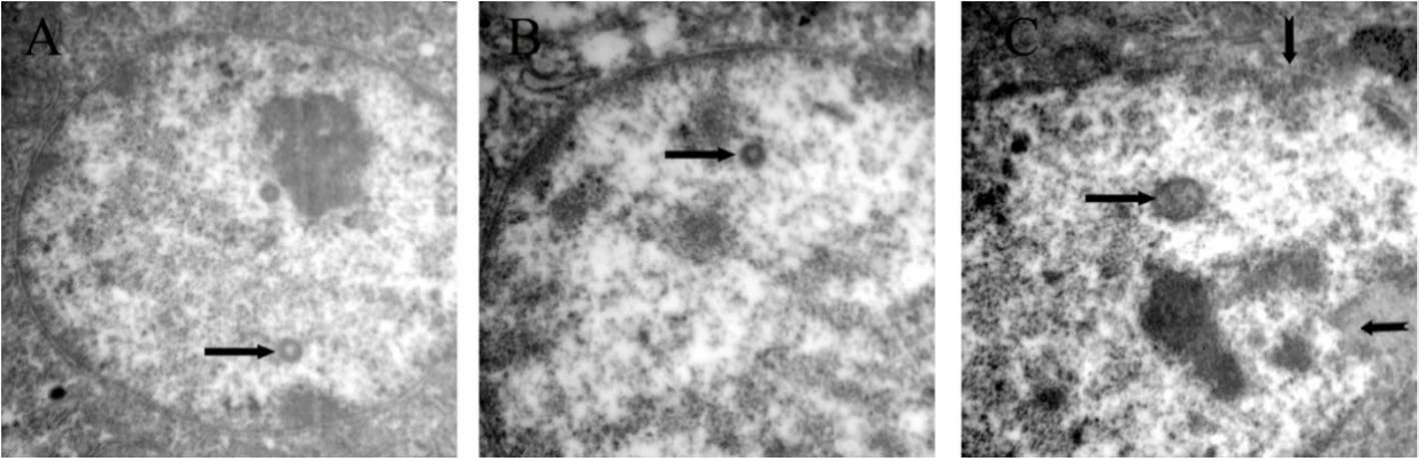
Electron microscopic examination of liver at 5 days post infection with fowl adenovirus showing different intra nuclear inclusion bodies (arrows), irregular nuclear membrane (notched arrow) (**a**) X1400 (**b**, **c**) X 1900

Electron microscopic examination of hepatic nucleus at 5 days post infection showed intranuclear inclusion bodies associated with irregular nuclear membrane in hepatic cells with proliferated virus particles (Fig. 7).

## Discussion

During the last 15 years, broiler cases with inclusion body hepatitis have been increased all over the world, resulting in major economic losses in poultry industry worldwide and most outbreaks have occurred in Egypt have been related to genotypes FAdV-D serotypes 2, 11, 1, 3 and 8a [17].

In this study, the most macroscopic lesions associated with collected field samples that suspected to be inclusion body hepatitis were pale, swollen livers with sub-capsular ecchymotic hemorrhages as described previously by [29]. Moreover, the histopathological examination showed characteristic lesions of FAdV represented in focal infiltration of mononuclear inflammatory cells, congestion of central vein (Fig. 1A), lytic necrosis of hepatic cells (Fig. 1B), appearance of basophilic Intranuclear inclusion bodies in hepatocytes (Fig. 1C) shape of intra nuclear inclusion bodies were dense basophilic inclusions which occupy most of the nucleus.These observations were partial similar to the previous studies which reported basophilic and eosinophilic INIB in hepatocytes in IBH infections [20].

Embryonic deaths occurred, after FAdV inoculation in SPF ECEs, within 5–10 days pi and hemorrhagic embryos, enlarged friable livers with yellow to reddish foci and/or diffuse greenish discoloration were detected (Fig. 2), these findings run in parallel with [34, 35].

Molecular detection of FAdVs s was performed through PCR assay using FAdV hexon L1-s and L1-as primers for amplification of the expected band 590 bp at position between nucleotides 301 and 890 that is type-specific domains in loop 1 of the hexon of fowl adenoviruses [36]. Hexon is the major gene of the adenovirus, and is known to have the charter of neutralizing epitope [26, 27], so sequencing of hexon gene is routinely method used for FAdVs serotyping. Phylogeny exposed that our strain belonged to fowl adenoviruses species D serotyped as FAdV-2 in addition to our strain is clustered with FAdVs Isolated from Europe, Egypt, Austria, Japan and Israel.

Pathogenicity studies have been done by oral inoculation of FAdV to 5 day-old White Leghorn Layer chicks, clinical IBH with necrotizing, hemorrhagic lesions and INIB in the liver were seen in all dead and clinically diseased euthanized birds following challenge with FAdV serotype –D oropharyngeally. Lesions severed in 5dpi including hepatitis and several pinhead white or red foci with mosaic appearance and clear fine edges of the liver and persisted to the end of experiment It is pointed out that many factors (virus strain—serotype, age at infection, younger chicks are more susceptible, and concurrent or previous infections affecting susceptibility or complicating the infection) may influence the course and severity of the disease [37] with 6% mortality that can be attributed to viremia occurring at 2 and 3 dpi.

Occurrence of IBH in young age layer chicks has been observed as a primary disease with no apparent association with immunosuppressive diseases such as IBD and CIA as mentioned by [38]. Oral route is the most possible path for developing the infection as reported by [39]. In the present study, oral inoculation of FadV-2 were efficient for developing the infection on 6 day old healthy broiler chicks and have led to similar signs to the natural infection. The incubation period varied from 6 to 15 days in naturally infections and from 48 to 72 h, occasionally up to 7 days experimentally [40] and the duration of the infection is generally reported as 2 or 3 weeks The examined chicks were positive for FAdV at 5 days of age with 6% mortality, as determined grossly in liver tissues, in typical cases livers were swollen, mottled and had articular pattern of fine linear and stellate subscapular hemorrhages. Necrotic foci have also been observed as showed by [16]. The persistence of gross lesions with the absence of deaths until the end of the experiment may confirm the ability of the virus to reproduce, even if it is in a small quantity.furthermore, in numerous cases the disease is subclinical unless complicated with other infectious agents, and signs and lesions are subtle.

Histopathological change of experimental birds infected with fowl adenovirus demonstrated the classical lesions of fowl adenovirus in the form of vacuolar degeneration of all hepatic tissue with appearance of eosinophilic intranuclear inclusion bodies, which confirmed by electron microscopic examination. Similar findings have been verified representing typical lesions of virulent fowl adenovirus in the liver [34, 41].

In addition, focal infiltration of inflammatory cells was noticed in different areas of hepatic tissue was recorded by [16] in fowl adenovirus -infected birds. In some cases, diffuse vacuolization of the lymphoid follicles beside severe lymphocytolysis in medullary lymphocytes were detected in some lymphoid follicles as previously seen in some studies [42]. Furthermore, fowl adenovirus infection results in immunosuppression in birds due to induction of sporadic apoptotic cells in lymphoid cells [43].

Moreover, pulmonary histopathological lesions characterized by congestion of numerous blood vessels and acute bronchopneumonia similar results were documented by [44] who detected that structural disorder of the pulmonary bronchus, inflammatory exudation, and alveolar rupture in infected birds.

## Conclusions

This study has detected the identification of FAdV-D serotype 2 by molecular and histopathological methods in a broiler flocks in Assiut province, Egypt as a primary disease without the need for a predisposing cause and/or immunosuppressive agents. Implementation of control measures and further work are crucial to detect prevalence of FAdVs infection in broiler chickens in Egypt. 

## Supplementary Information

**Additional file 1.** 

**Additional file 2:** **Supplementary Table 1.** Representing the history of suspected sample infected, farmflocks, with FAdV (IBH) including strain used in the pathogenicity study.

## Data Availability

The datasets supporting the conclusions of this article are included within the article. The datasets used and/or analyzed during the current study available from the corresponding author on reasonable request.

## Glossary

FAdVs: Fowl Adenoviruses
RFLP: Restriction fragment length polymorphism
IBH: Inclusion body hepatitis
HHS: Hepatitis-hydro pericardium syndrome
GE: Gizzard erosions
INIB: Intra nuclear inclusion bodies
NBF: Neutral buffered formalin
PBS: Phosphate buffered saline
PCR: Polymerase chain reaction
ECEs: Embryonated chicken eggs
NCBI: National Center for Biotechnology Information
OIE: World Organization for animal health
TCID50: 50% tissue culture infective dose
dpi: day post infection
TEM: Transmission Electron microscopy
