# Supplementary figures and images for: Genotyping and pathogenicity of fowl adenovirus isolated from broiler chickens in Egypt
Source: BMC Vet Res. 2022 Aug 30;18:325. doi: 10.1186/s12917-022-03422-1 (PMC9425993; doi:10.1186/s12917-022-03422-1)

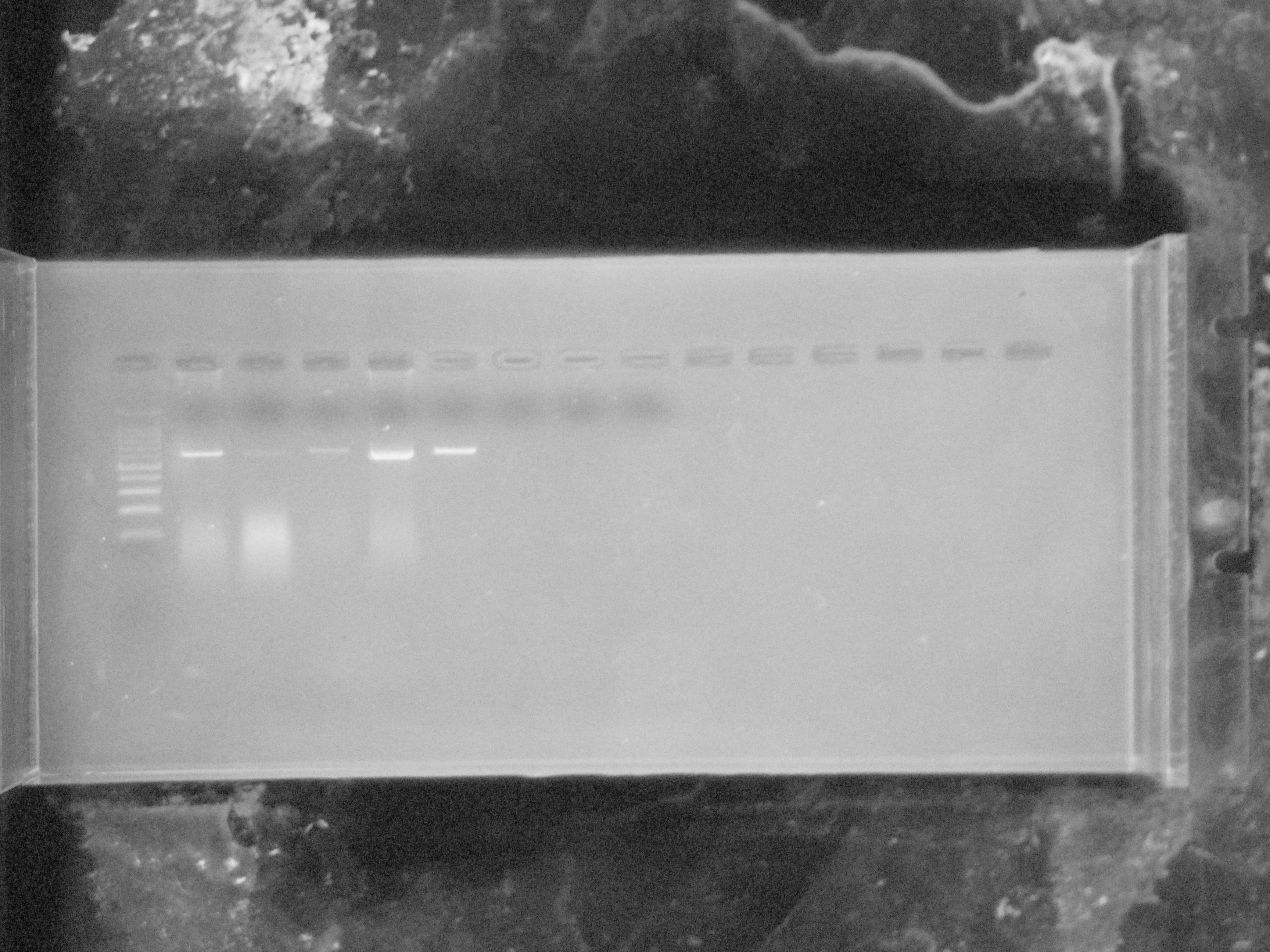

Supplement: Supplementary file 1 — Additional file 1. [file 12917_2022_3422_MOESM1_ESM.jpg]
