# Supplementary material for: Genotyping and pathogenicity of fowl adenovirus isolated from broiler chickens in Egypt
Source: BMC Vet Res. 2022 Aug 30;18:325. doi: 10.1186/s12917-022-03422-1 (PMC9425993; doi:10.1186/s12917-022-03422-1)
Supplement: Supplementary file 2 — Additional file 2: Supplementary Table 1. Representing the history of suspected sample infected, farmflocks, with FAdV (IBH) including strain used in the pathogenicity study. [file 12917_2022_3422_MOESM2_ESM.docx]

| **Presence of**  **Hexon gene by PCR** | **No. of**  **collected liver samples** | **Postmortem**  **Lesions** | **Vaccination** | **Mortality**  **rate** | **Age** | **Flock No.** |
| --- | --- | --- | --- | --- | --- | --- |
| +ve | 10 | **Pale and swollen liver** | Against AI,ND and IBD | 3% | 30 days | **1** |
| +ve | 10 | **Pale and swollen liver with subscapular hemorrhage** | Against AI,ND and IBD | 13% | 22 days | **2** |
| +ve | 10 | **swollen liver with greenish discoloration** | Against IB ,ND and IBD | 6% | 28 days | **3** |
| +ve  **Used for pathogenicity (OK482670)** | 10 | **Pale and swollen liver with subscapular hemorrhage** | Against AI,ND and IBD | 10% | 25days | **4** |
| -ve | 10 | **Friable and swollen liver** | Against AI,ND and IBD | 7% | 29 days | **5** |
| -ve | 10 | **Pale and swollen liver** | Against AI and ND | 6% | 34 days | **6** |
| -ve | 10 | **Swollen liver with subscapular hemorrhage** | Against IB,ND and IBD | 5% | 28 days | **7** |
| -ve | 10 | **Pale and swollen liver** | Against AI and ND | 8% | 30 days | **8** |
| -ve | 10 | **Pale and friable liver** | Against ND and IBD | 4% | 33 days | **9** |
| -ve | 10 | **Pale and swollen liver** | Against ND and IBD | 6% | 37days | **10** |

Table 1: representing the history of suspected sample infected, farm flocks, with FAdV (IBH) including strain used in the pathogenicity study.
